# Using a Distant Abdominal Skin Flap to Treat Digital Constriction Bands: A Case Report for Vohwinkel Syndrome

**DOI:** 10.1097/MD.0000000000002762

**Published:** 2016-02-12

**Authors:** Mingzi Zhang, Kexin Song, Ning Ding, Chang Shu, Youbin Wang

**Affiliations:** From the Department of Plastic Surgery, Peking Union Medical College Hospital, Beijing (MZ, KS, YW); Department of Neurosurgery, Qingdao Huangdao District Hospital of Traditional Chinese Medicine, Qingdao, Shandong (ND); and Department of Dermatology, Peking Union Medical College Hospital, Beijing, China (CS).

## Abstract

In this study, a Vohwinkel syndrome case is presented where in 5th digit constriction bands in the right hand were reconstructed using a distant abdominal skin flap. Vohwinkel syndrome, or keratoderma hereditarium mutilans, is a rare, autosomal dominant genetic skin condition that causes palmoplantar hyperkeratosis and constricts finger and/or toe bands. In a typical manifestation, the finger and toe constriction bands lead to progressive strangulation and autoamputation, which requires immediate clinical treatment. Topical keratolytics and systemic retinoids have been used to treat hyperkeratosis but without consistent results. Only 1 effective approach for autoamputation has been accepted, reconstructive surgery.

Applying a distant abdominal skin flap produced satisfying postoperative effects at the 18-month follow-up.

## INTRODUCTION

Vohwinkel syndrome, or keratoderma hereditarium mutilans, is a rare, autosomal dominant genetic skin condition.^1^ The syndrome is manifested by palm and sole hyperkeratosis, which creates a typical honeycomb pattern and starfish appearance on extensor surfaces. In a typical manifestation, the finger and/or toe constriction bands begin with the 5th digit, which leads to progressive strangulation and autoamputation. Modern genetics show that Vohwinkel syndrome results from 2 mutations, in the gap junction beta-2 gene coding connexin-26 and in the loricrin gene. The gap junction beta-2 gene is associated with classic Vohwinkel syndrome. Patients present palm and sole hyperkeratosis, constriction bands at the digits and a starfish appearance on the dorsal portions of the hands and feet. Other characteristics of this mutation include alopecia, nail abnormalities, and hearing loss.^2^ The loricrin gene protein plays a paramount role in the forming the cornified cell envelope. Sequential loricrin deposition increases the envelop thickness and rigidity. Patients with this mutation exhibit palmoplantar keratoderma, hyperkeratotic knuckle pads on the dorsal portion of the fingers, and constriction bands around the fingers and/or toes.^3,4^

Topical keratolytics and systemic retinoids have been used to treat hyperkeratosis but without consistent results.^5^ Only 1 effective approach for autoamputation has been accepted reconstructive surgery. Because Vohwinkel syndrome is a rare genetic skin disease, reference reports on surgical treatments that mention Z-plasty, cross-finger flaps, and skin grafting are limited. In this study, a case of Vohwinkel syndrome is presented where in constriction bands at the 5th digit on the right hand were reconstructed using a distant abdominal skin flap. Thereafter, an 18-month follow-up was performed to evaluate the postoperative results.

## CASE REPORT

**FIGURE 1:**
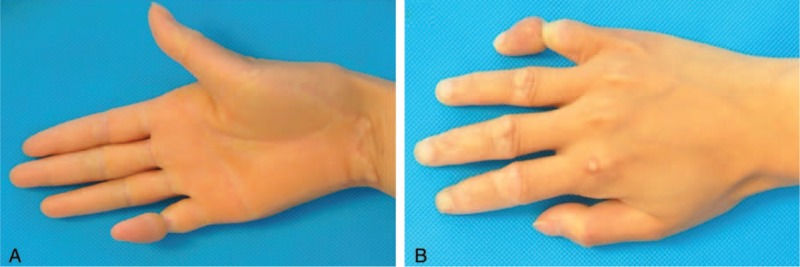
(A) Right palm hyperkeratosis, (B) the constriction band on the 5th finger of the right hand severely congested blood flow to and from the distal portion of the finger.

A 24-year-old female patient visited our outpatient clinic in January, 2014 because she suffered from an ainhum-like constriction band on the 5th finger of her right hand. A physical examination revealed hyperkeratosis in both palms (Figure 1A) and soles, constriction bands at the 5th right finger, and hyperkeratotic knuckle pads on the dorsal aspects of both hands (Figure 1B). The palm and sole hyperkeratosis began with no predisposition when she was 6 years old. The constriction bands at the right 5th finger began at the same time but progressed slowly and slightly. One year ago, the distal little finger beyond the constriction band produced pain and became more serious in cold weather with the patient's skin color slowly but progressively becoming cyanotic. This study does not involve ethic issues or patient privacy consent; therefore, no ethics approval or informed consent is necessary.

**FIGURE 2:**
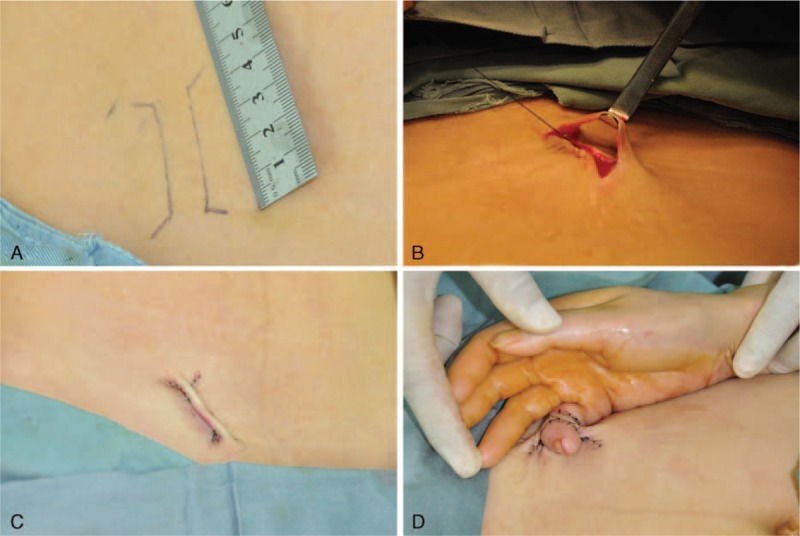
Surgical procedures: (A) Bridge-shaped skin flap on the abdomen of 5 × 1 cm was designed according to the constriction length. (B, C) Separated skin flap and sutured bottom incision. (D) The skin flap covered the constriction wound after excision.

**FIGURE 3:**
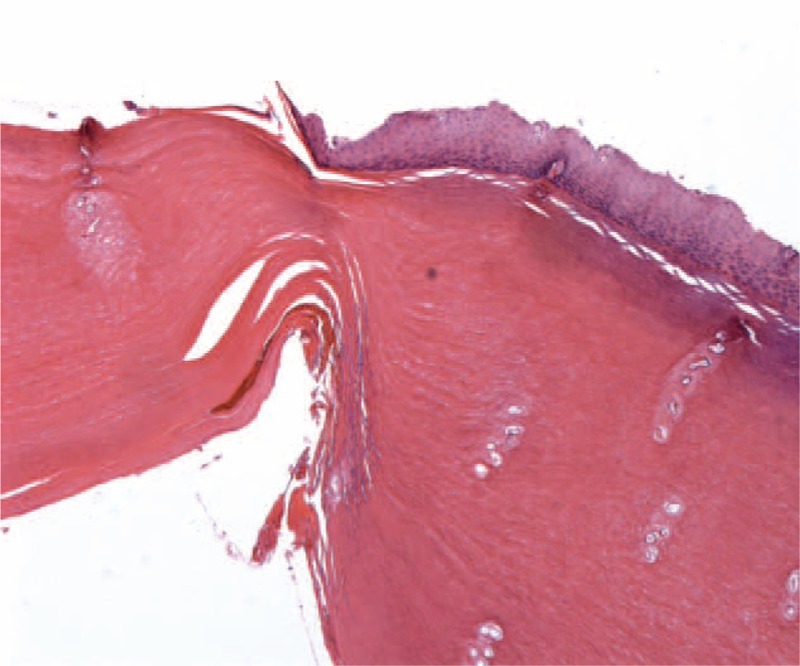
Histopathological examination of the constriction band. The keratin increased significantly.

Based on this patient's condition, a surgical approach was designed for the 5th digit of the right hand. The constriction was corrected using a distant abdominal skin flap under local anesthesia. First, we designed a 5 × 1 cm bridge-shaped skin flap on her abdomen following the length of the constriction band (Figure 2A). The skin flap position should be appropriate for the patient's comfort after surgery. Next, the marked line was incised, the skin flap was raised, and the bottom incision was sutured (Figure 2B and C). After removing half of the constriction, the skin flap should cover the wound and be properly dressed (Figure 2D). The removed constriction band was collected for a histopathological examination (Figure 3). After 3 weeks, we performed a clipping test on the top pedicle of the flap. Blocking the blood to 1 pedicle for at least 1 hour does not remarkably affect the blood supply and is the proper time to cut this pedicle. The interval between cutting these 2 pedicles should be at least 3 weeks to ensure that a stable blood supply is established between the recipient bed and distant abdominal skin flap. In the last operation, the other half of the constriction band should be removed, and the excessive skin flap should cover the wound properly. Until the last surgery, the patient's right hand was fixed to her abdomen; the patient should be instructed to protect the skin flap by avoiding abrupt and violent movement. The ward temperature and humidity remained at 18 to 22 °C and 50% to 60% RH. The dressing was changed every day. After removing the dressing, the color and flap condition should be observed and judged. Next, physiological saline was used to clean the wound. The site was disinfected through embrocating with iodophor 3 times and covering the site with a new dressing. After each surgery, the patient received a hyperbaric oxygen treatment to improve flap survival. No other particular care was provided for this patient.

**FIGURE 4:**
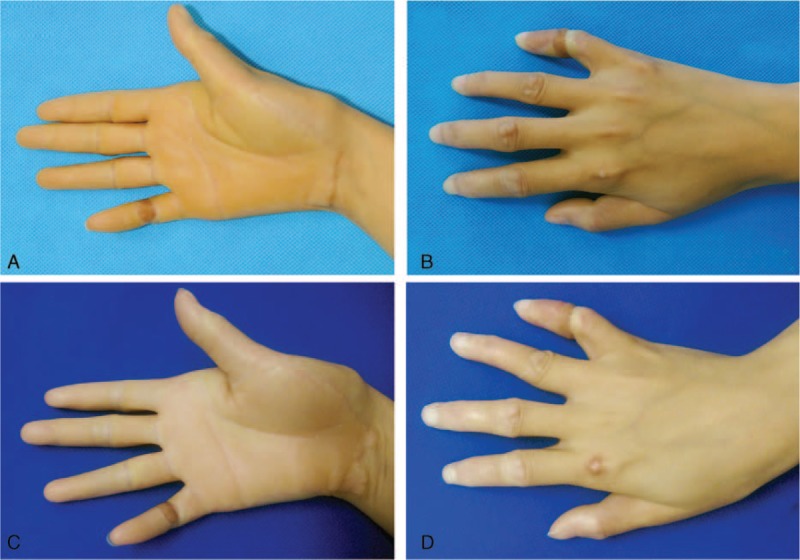
(A, B) A 12-month follow-up after surgery. (C, D) An 18-month follow-up after surgery. The constriction band was replaced with an abdominal skin flap, after which no congestion was observed.

This patient visited our outpatient practice regularly, and no complications have been observed. In the latest follow-up at 18 months after the operation, the patient felt satisfied with the operation effects, and no constriction band has reoccurred (Figure 4).

## DISCUSSION

Vohwinkel syndrome is a rare, autosomal dominant dermatosis and was first described in 1929 by Vohwinkel. It is a syndromic form of diffuse palmoplantar keratoderma that manifests as palm and sole hyperkeratosis with a honeycomb appearance and constriction bands on the digits. Through 2013, approximately 50 cases were reported in the literature.^6^ The mechanism underlying Vohwinkel syndrome has not been fully explored. In recent studies, 2 mutations were identified in Vohwinkel syndrome: a mutation in the gap junction beta-2 gene that codes connexin-26 and in the loricrin gene. Clinically, this condition manifests in infants and becomes more obvious in adulthood. Constriction fibrous bands typically appear at the 5th digit and can lead to progressive strangulation and autoamputation, which is the most immediate threat and requires surgical treatment. Starfish-shaped keratosis is characteristic of this rare disease and may appear on the elbow, knee joint, and dorsal portion of the hands and feet. Other associated symptoms include alopecia, hearing loss, spastic paraplegia, myopathy, ichthyosiform dermatosis, and nail abnormalities.^7–9^ This patient was diagnosed based on her clinical appearance and dermatologist consultations. The differential diagnosis for Vohwinkel syndrome includes Olmsted syndrome, acral keratoderma, congenital pachyonychia, Sybert's palmoplantar keratoderma, Meleda disease, and Gamborg–Nielsen palmar and plantar keratoderma, all of which are a keratoderma associated with autoamputation of the digits.^10^ Nonhereditary diseases that may cause constricting bands include leprosy, tertiary syphilis, frambesia, ainhum, scleroderma, amniotic bands, ergotamine poisoning, and Reynold's syndrome.^10^

Various forms of medical treatment have been used for Vohwinkel syndrome. Vitamin A derivatives, such as etrinate^11^ and retinoic acid,^12,13^ have been particularly and unpredictably successful. However, the constriction band that produces progressive strangulation and autoamputation can only be resolved through surgical methods. Surgical approaches to constriction bands were studied by Weriede^14^ and Luk et al^15^ in 1984 and 1986. Z-plasty, cross-finger flaps, and skin grafting are the most common surgeries used for constriction band; no reports mention using a distant skin flap. However, these techniques exhibit temporary success but long-term failure.^16^ The longest follow-up was 3 years in a patient treated with Z-plasty. A cross-finger flap was successful at an 18-month follow-up.^16^ According to Liebman's theory, excising constriction bands is not sufficient, and the condition will recur over time considering the characteristics of the disease and its tendency toward specific areas of the digits.^16^ Skin grafting tends to maintain the donor site characteristics. Therefore, skin grafting may permanently solve the problem.

Based on these reports, we designed a surgical approach using a distant abdominal skin flap to repair the wound after excising a constriction band from digits. The abdominal skin flap was used for the following reasons.

Using normal skin tissue on abdomen maybe appropriate for permanently solving the band recurrence problem.

In this therapy, local immobilization is important for obtaining a desired result. The local immobilization time is relatively long; therefore, fixing this patient's right hand to her abdomen would not affect her daily life much.

Fat tissue is rich in the abdomen. The skin flap we used contains full-thickness skin and tissue with little fat. A skin flap with some fat tissue may improve appearance effect after a grafting surgery.

After surgery, the donor site scar can be easily hidden by clothing.

Compared with former techniques, using a distant abdominal skin flap can ensure blood supply and flap survival as well as improve appearance and may permanently solve the constriction band recurrence problem. Our distant abdominal skin flap grafting treatment was successful with no evidence of recurrence at the 18-month follow-up. However, this technique requires a long treatment period, 3 operations under local anesthesia, and a relatively long local immobilization time, which could inconvenience a patient.

Applying a distant abdominal skin flap is optional for treating digital constriction bands in Vohwinkel syndrome patients, and we reveal satisfactory postoperative effects after an 18-month follow-up. Using a distant skin flap is promising, but further follow-up is necessary to ensure that the condition does not recur.
